# Biocompatibility of a polymer based on Off-Stoichiometry Thiol-Enes + Epoxy (OSTE+) for neural implants

**DOI:** 10.1186/s40824-015-0041-3

**Published:** 2015-09-21

**Authors:** Fredrik Ejserholm, John Stegmayr, Patrik Bauer, Fredrik Johansson, Lars Wallman, Martin Bengtsson, Stina Oredsson

**Affiliations:** Department of Biomedical Engineering, Lund University, Box 118, Lund, 221 00 Sweden; NeuroNano Research Centre, Lund University, Medicon Village, Scheelevägen 8, Lund, 223 81 Sweden; Department of Laboratory Medicine, Lund University, Box 118, Lund, 221 00 Sweden; Department of Biology, Lund University, Box 118, Lund, 221 00 Sweden

**Keywords:** Biocompatibility, In Vitro toxicity, LC-MS, Neural implant, Polymer, Off-Stoichiometry Thiol-Enes + EpoxyOSTE+

## Abstract

**Background:**

The flexibility of implantable neural probes has increased during the last 10 years, starting with stiff materials such as silicone to more flexible materials like polyimide. We have developed a novel polymer based on Off-Stoichiometry Thiol-Enes + Epoxy (OSTE+, consisting of a thiol, two allyls, an epoxy resin and two initiators), which is up to 100 times more flexible than polyimide. Since a flexible neural probe should be more biocompatible than a stiff probe, an OSTE+ probe should be more biocompatible than one composed of a more rigid material. We have investigated the toxicity of OSTE+ as well as of OSTE+ that had been incubated in water for a week (OSTE+H_2_O) using MTT assays with mouse L929 fibroblasts. We found that OSTE+ showed cytotoxicity, but OSTE+H_2_O did not. Extracts were analyzed using LC-MS and GC-MS in order to identify leaked chemicals.

**Results:**

Most constituents were found in extracts of OSTE+, whereas only initiators were found in OSTE+H_2_O extracts. The detected levels of each chemical found in the LC-MS and the GC-MS analysis were below the toxicity level when compared to MTT assays of all the individual chemicals, except for one of the initiators that had an IC_50_ value close to the detected levels.

**Conclusion:**

Our notion is that the toxicity of OSTE+ was caused by one of the initiators, by impurities in the constituents or by synergistic effects of low doses of leaked chemicals. However, our conclusion is that if OSTE+ is incubated for one week in water, OSTE+ is not cytotoxic and suitable for further *in vivo* studies.

## Background

The current research on neural probes for deep brain stimulation has created new possibilities to treat conditions such as chronic pain and motor symptoms associated with Parkinson’s disease, in addition to enabling control of prosthetic limbs [1–4]. During the last couple of years, the development of implantable neural probes have been oriented towards flexible probes [5–14] made out of polymers, instead of the previously stiff probes made of silicon [15]. Since a more flexible probe will reduce the strain at the interface of the probe and the tissue when compared to a more rigid probe, it should also reduce the tissue response to the probe, thus a more flexible neural probe should be more biocompatible then a more rigid probe [13, 16]. Currently, the choice of material for both insulation as well as for the backbone of flexible intra-cortical neural probes has been Parylene C or polyimide, and for flexible cortical neural probes: either Parylene C, polyimide or polydimethylsiloxane (PDMS). When comparing the flexibility according to Young´s modulus, where a more rigid material has a higher Young’s modulus value and a more flexible material has a lower value (Table 1), PDMS is more flexible than Parylene C and polyimide, even though all three materials have significant stiffness as compared to brain tissue. However, a technical drawback with PDMS is the difficulty in patterning it using UV-lithography, and thus it has to be molded, impairing the possibility of producing thin probes. In addition, a thin PDMS-based probe would be so fragile that it would likely probably not be possible to implant it into brain tissue.

**Table 1:** Young´s modulus values of brain tissue, Parylene C, PDMS, polyimide and OSTE+ [12–15]

Material	Young’s modulus
Brain tissue	0.5-1 kPa
Parylene C	4 GPa
PDMS	0.36–1.24 MPa
Polyimide	2.48 GPa
OSTE+ @ 10 °C	1.9 GPa
OSTE+ @ 37 °C	50 MPa

Off-Stoichiometry Thiol-Enes + Epoxy (OSTE+) is a new polymer that was developed to be used in microfluidic devices [17] instead of PDMS, and we have then further developed it into a potential material for intra-cortical neural probes [18]. The Young´s modulus of OSTE+ can be tuned by shifting the ratio between the constituents and it can easily be patterned using UV-lithography. We have developed a composition of OSTE+ that is as stiff as polyimide at 10 °C, and roughly 100 times more flexible at a physiological temperature, 37 °C (Table 1) [18]. In UV-lithography, OSTE+ is a negative photoresist and can be spin-coated into very thin layers where the thickness of the layer can be controlled by the spin speed. Another advantage OSTE+ affords is that the fabrication protocol is close to that of polyimide and it can therefore easily replace polyimide in current neural probes.

There are several studies showing the biocompatibility of Parylene C, PDMS and polyimide [19–23]. However, since OSTE+ is a new material, limited work has been done in this area [24].

In this study, our goals were to determine the biocompatibility of OSTE+ as well as investigate if there was leaching of constituents from the final polymer. The biocompatibility of various preparations of OSTE+ was tested using *in vitro* cytotoxicity assays (MTT assays) together with microscopy techniques and the analysis of possible constituents leaching from the materials was performed with liquid chromatography - mass spectroscopy (LC-MS) and gas chromatography – mass spectroscopy (GC-MS). This is an initial study of the cytotoxicity of OSTE+ and the results show that OSTE+, if handled correctly, renders low toxicity to cells, this warrants further studies in compliance with ISO 10993–1 in order to completely deduce the biocompatibility.

## Methods

**Fig. 1:**
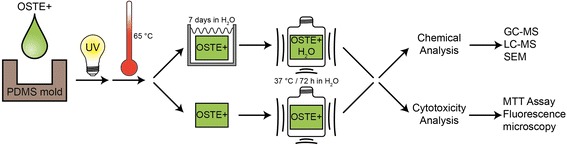
A schematic representation of the procedure used in this study. A mixture of OSTE+ was shaped into blocks using a PDMS mold. The OSTE+ material was formed by a two-step polymerization process: first a UV-initiated step (exposure time of 40 min) followed by a heat-initiated step in which the samples were placed in an oven at 65 °C overnight. Half of the samples were placed in water for a week, allowing eventual unreacted constituents to leak out. Extractions were made by placing a sample of each material into a vial with water and the vial was then gently shaken at 37 °C for 72 h. The extractions were then used in chemical analysis and biological assays

The manufacturing of the OSTE+ samples started with a molding step where the samples were cured in a PDMS mold using UV-light, followed by a post exposure bake at 65 °C. Half of the OSTE+ samples were then incubated in MilliQ water (Millipore, Bedford, MA, USA) at room temperature for 7 days to allow non-bound constituents to leach out. These samples are called OSTE+H_2_O throughout this paper. All samples were then incubated in water at 37 °C for 72 h (in compliance with ISO standard 10993–12) and the extractions were used in two different assay systems: chemical analysis and cytotoxicity analysis (in compliance with ISO standard 10993–5). The whole process is shown schematically in Fig. 1.

The cytotoxicity tests were performed using MTT assays in compliance with ISO standard 10993–5, and all samples were extracted using ISO standard 10993–12.

### Polymer fabrication

#### OSTE+

OSTE+ is produced in a two-step polymerization reaction as previously described [18]. First, a fast UV-initiated radical polymerization step results in a cross-linked network of some of the thiol groups and of all the ene groups in the allyls. Then a thermal anionic polymerization step results in a fully polymerized network with the unreacted thiol groups and the epoxy resin.

In the first polymerization step, UV light was used o activate Lucirin® TPO-L (BASF, Germany) that initiated cross-linking between the thiol, tris[2-(3-mercaptopropionyloxy)ethyl] isocyanurate (90 %, Sigma Aldrich, Germany), and the two allyls, trimethylolpropane diallyl ether (90 %, Sigma Aldrich) and 2,4,6-triallyloxy-1,3,5-triazine (97 %, Sigma Aldrich). In the second polymerization step, 1,5-diazabicyclo[4.3.0]non-5-ene (98 %, DBN, Sigma Aldrich) was used as the initiator for cross-linking between the thiol and the epoxy resin, D.E.N 341 Epoxy Novolac resin (Dow Chemicals, USA).

All ingredients were weighed according to a stoichiometric ratio of monomers of 1.5 : 0.47 : 0.53 : 0.5 for thiol, diallyl, triallyl and the epoxy resin, respectively. TPO-L and DBN were used at 0.2 %. Finally, 3 % acetone (Sigma Aldrich) was added to the mixture. All ingredients were mixed and degassed under vacuum for 5 min. The mixture was then poured into a pre-fabricated PDMS mold. The mold containing the mixture was then covered with a polycarbonate film and exposed to UV-light for 40 min using a Karl Suss MA4 mask aligner. The polycarbonate film was removed after the exposure and the OSTE+ pieces were hard-baked (to finalize the thiol-epoxy polymerization) in an oven at 65 °C overnight. The ratio of the different constituents results in an OSTE+ with a glass transition temperature of 39 °C [18].

#### Polyimide

Polyimide was selected as a reference material since it is a material used in neural probes today [8, 9, 11, 13, 20, 22]. Polyimide (Durimide 7505, FujiFilm, Belgium) was poured into a PDMS mold, followed by a 2 h soft-bake (to remove the solvents) at 85 °C. The mold with polyimide was exposed to UV-light for 40 min using a Karl Suss MA4 mask aligner. The polyimide was then hard-baked (cured) in an oven at 200 °C for 4 h. Finally, the polyimide pieces were rinsed in MilliQ water.

#### HDPE

A sheet of HDPE (Bay Plastics LTD, England) was cut into pieces using a milling machine. The pieces were cleaned with acetone (Sigma Aldrich) in an ultrasonic bath followed by a rinse in ethanol (Solveco, Sweden) and MilliQ water.

#### Polymer extraction

Extractions of polymer samples were obtained according to ISO standard 10993–12. The extraction was performed in cleaned borosilicate glass vials and water (LC-MS Ultra Chromasolv, Sigma Aldrich, for the chemical analysis and MilliQ water for the cytotoxicity analysis) was used as the extraction medium. The ratio of the surface area of the OSTE+, OSTE+H_2_O, polyimide or HDPE samples and the water was 3 cm^2^/ml. The samples were washed in MilliQ water before extraction. The vials were gently shaken at 37 °C for 72 h. When the extraction solution was used in cytotoxicity tests, the polymer samples were first sterilized using an autoclave at 121 °C and the extractions were performed with sterile water.

### Chemical analysis

#### LC-MS analysis

To obtain standards for the LC-MS analysis, an exact amount of each chemical used for the production of OSTE+ was dissolved in methanol (Sigma Aldrich) overnight (except acetone and D.E.N 431). The following day the solutions were diluted to a concentration of 500 μg/ml using 50 % MilliQ water and 50 % methanol. This was followed by a second dilution step to 50 μg/ml using MilliQ water. Acetone was not tested since it is not detectable in the LC-MS system used; D.E.N 431 was not tested since it is not water-soluble. To simulate the UV-lithography process of the fabrication of OSTE+, all standards were exposed to UV-light using a Karl Suss MA4 mask aligner for 40 min. All standards, extractions (described above) of OSTE+, extractions of OSTE+H_2_O as well as blank samples, were analyzed using LC-MS.

A LC-MS system (QStar XL, Sciex, together with an Agilent 1100 LC system) was used with an Acquity CHS C18 1.7 μm 2.1x50 mm column and a sample volume of 5 μl. The mass spectrometer was configured to scan 120–1000 Da at 2 scans/s. Acetonitrile (LC-MS, Chromasolv, Sigma Aldrich) and MilliQ water were used at a flow rate of 250 μl/min. The concentration of acetonitrile in the mobile phase was: starting at 5 % for 1 min, ramp to 95 % over 3 min, hold for 2 min, ramp down to 5 % in 0.1 min.

#### GC-MS analysis

To obtain standards for the GC-MS analysis, an exact amount of all constituents (except acetone) was dissolved in toluene (Sigma Aldrich and VWR). To simulate the UV-lithography process of the fabrication of OSTE+, all samples were exposed to UV-light for 40 min using a Karl Suss MA4 mask aligner. 400 μl of the OSTE+ extractions or OSTE+H_2_O extractions (described above) was pipetted to a new vial containing 1 ml of toluene. The toluene extraction samples were mixed using a vortex for 40 h, followed by centrifugation for 1 min. To ensure that all chemicals in the extracts were transferred into the organic phase, 2 samples of each extraction were made acidic by adding 50 μl of 0.1 M HCl (VWR) and 2 samples of each extraction were made basic by adding 50 μl of 0.1 M NaOH (VWR) just before the addition of toluene to each extraction. The extracts spiked with HCl are referred to as OSTE+/HCl and OSTE+H_2_O/HCl throughout the paper and the extracts spiked with NaOH are referred to as OSTE+/NaOH and OSTE+H_2_O/NaOH throughout the paper. All samples and extracts together with a sample of pure toluene were analyzed using GC-MS. Acetone was not tested since it is used as a cleaning step in the GC-MS analysis.

All analyses were performed on a gas chromatograph (Agilent 6890) equipped with an injector in split mode at 250 °C, equipped with an auto sampler (sample volume of 5 μl) and a mass spectrometry detector (Agilent 5973-N). The column used in the system was a HP-5 ms fused silica capillary column (5 % phenyl-methylpolysiloxane) of 30 m × 0.25 mm with a phase thickness of 0.25 μm. The temperature program of the column was: 50 °C, hold for 1 min, ramp to 300 °C over 10 min, hold for 15 min. The carrier gas used was helium at a flow rate of 1.2 ml/min. The mass spectrometer was run in either scan mode (30–600 Da with 2.6 scan/s) or in selected ion monitoring (SIM) mode, using mass to charge ratios (m/z) of 70, 81, 82, 99, 123, 125, 147, 187, 197, 254, 321 together with a dwell time of 20 ms.

#### Scanning electron microscopy analysis of OSTE+

Four OSTE+ pieces were cut into two halves. One half was put into MilliQ water for 7 days and then each pair was studied by scanning electron microscopy (SEM, SU 8010, Hitachi, Japan) to see if 7 days of incubation in water affected the surface of OSTE+.

### Cytotoxicity analysis

Four different materials were tested in the MTT assay: OSTE+, OSTE+H_2_O, polyimide and HDPE. Polyimide was selected since it is regarded as biocompatible and there are several studies that can be used for comparison. HDPE is a biocompatible material that sometimes is used as a negative control in different cell viability assays [22, 25, 26].

#### Cell culture

The mouse fibroblast cell line L929 was purchased from American Type Culture Collection (ATCC, LGC standards AB, Borås, Sweden) and was grown as a monolayer culture in complete cell culture medium, *i.e.* RPMI-1640 medium supplemented with 10 % fetal bovine serum (FBS), 100 U/ml penicillin, 100 μg/ml streptomycin and 1 mmol/l L-glutamine, at 37 °C in 5 % CO_2_ in air. The cells were sub-cultured twice every week at a density of 12 500 cells/cm^2^.

#### Polymer extractions for cell culture

The extracts were obtained as described above and kept at 4 °C for a maximum time of 24 h before use in the cytotoxicity assay with L929 cells. The extracts were diluted 1:2 in 2x concentrated RPMI-1640 medium supplemented with 20 % FBS, 200 U/ml penicillin, 200 μg/ml streptomycin and 2 mmol/l L-glutamine, *i.e.* a final extract concentration of 50 % in 1x concentrated RPMI-1640 containing 10 % FBS, 100 U/ml penicillin, 100 μg/ml streptomycin and 1 mmol/l L-glutamine, before addition to cell cultures Further dilutions of the 50 % concentrated polymer extracts were done in 1x concentrated RPMI-1640 medium supplemented with 10 % FBS, 100 U/ml penicillin, 100 μg/ml streptomycin and 1 mmol/l L-glutamine.

#### MTT assay – extract testing

L929 cells were seeded in complete cell culture medium in 96-well plates (2000 cells/well in 180 μl medium) and were then incubated at 37 °C in 5 % CO_2_ in humidified air for 24 h. The medium was then removed and changed to medium containing the different extracts at concentrations ranging from 6.25–50 % medium containing 2.5 % dimethyl sulfoxide (DMSO: positive control) or control medium (complete cell culture medium with 0 % extract). The cells were then kept at 37 °C in 5 % CO_2_ in humidified air for 72 h before addition of MTT to a final concentration of 0.5 mg/ml. The 96-well plates were incubated with MTT at 37 °C for 1 h before removal of the medium, and the cells with blue formazan crystals were then dissolved in DMSO. The following spectrometry reading at 540 nm was done using a SPECTRAmax M2 instrument (Molecular Devices, Sunnyvale, CA, USA) and analyzed using SoftMax® Pro v. 4.6 (Molecular Devices). A number of 3–6 biological replicates (*i.e.* the testing of independent extracts of OSTE+, OSTE+H_2_O, polyimide and HDPE) were used for all the treatments of the L929 cells and a number of 6 technical replicates (*i.e.* number of n) from each extract were used for each biological replicate. After correcting all absorbance values for background, the percent of control was calculated as absorbance units in the presence of test compound as percentage of that in control. The results of the MTT assay are resumed to reflect the cell number, thus, 50 % percent MTT reduction of control implies that there were 50 % less cells at that treatment concentration [27].

#### MTT assay - individual chemicals

Each chemical used in the fabrication (except acetone), 1 or 0.1 mg, was added to a sterile flask and 1000 μl sterile water was added. Further dilutions were made using sterile water. D.E.N 431 was not soluble in water and after shaking the sample and letting it stand for a while, only the water solution was used in the test. L929 cells were seeded in complete medium in 96-well plates (2000–3000 cells/well in 180 μl medium) and the plates were incubated in a CO_2_ incubator for 24 h before addition of the chemicals. Indicated concentrations of the chemicals were added to the wells and the cells were incubated for 72 h at 37 °C in 5 % CO_2_ in humidified air. The dose response was evaluated using MTT as described above.

#### Phase contrast and fluorescence microscopy

L929 cells were seeded in 12-well plates containing sterile glass coverslips, whereupon they were incubated at 37 °C in 5 % CO_2_ in humidified air for 24 h. The growth medium was then removed and changed to growth medium without extract or containing the different polymer extracts (at a concentration of 50 %, obtained as described above). The cells were then incubated for 72 h before they were photographed with an Olympus CKX41 inverted phase contrast microscope (Olympus Corporation, Tokyo, Japan) equipped with an Olympus SC30 camera (Olympus Corporation) then analyzed using the software cellSense Standard version 1.4 (Olympus Corporation). The cells were then fixed in 3.7 % paraformaldehyde for 15 min and washed with phosphate-buffered saline (PBS). The fixed cells were stained with Alexa Fluor 647-conjugated phalloidin in PBS containing 1 % bovine serum albumin and 1 % Tween-20 for 1 h and in PBS containing 1 μg/ml bisbenzimide for 1.5 min to visualize the cytoskeleton and nuclei, respectively. The glass coverslips were mounted on glass slides using Mowiol 4–88 and left in a refrigerator overnight. The samples were imaged with a LSM510 confocal laser scanning microscope (Carl Zeiss Microscopy GmbH, Oberkochen, Germany) equipped with a Hamamatsu R6357 photomultiplier (Hamamatsu Photonics K.K., Hamamatsu, Japan). A 405 nm diode-pumped solid-state laser was used to excite the bisbenzimide and a band-pass filter of 420–480 nm was used for the emission. The Alexa Fluor 647-conjugated phalloidin was excited with a 633 nm HeNe ion laser and a 650 nm long-pass filter was used for the emission. The cells were visualized by z-stacks composed of single optical planes in high magnification and the images were analyzed with the software ZEN 2012 (black edition) version 8.0 (Carl Zeiss Microscopy GmbH).

## Results

### Chemical analysis using LC-MS

Standards of all constituents (except acetone and D.E.N 431), extracts of OSTE+, extracts of OSTE+H_2_O and blank samples were analyzed using LC-MS.

From the total ion current (TIC) chromatogram together with the molecular structure of each chemical and the mass to charge ratio the lower limit of detection (LOD) for each chemical could be calculated as well as the detection level for each chemical in the samples (Table 2). The LOD was calculated using a 3:1 ratio between signal/noise. Assuming that Beer Lambert’s law was fulfilled, there should be a linear relationship between the recorded signal and the concentration. Then it was possible to calculate the detected concentration using a single-point calibration curve according to the following equation [28, 29]:

$$ \mathrm{C}=\frac{{\mathrm{C}}_{\mathrm{ref}}}{{\mathrm{h}}_{\mathrm{ref}}}\mathrm{h} $$

where C is the detected concentration, C_ref_ is the concentration of the standard, h_ref_ is the peak height of the standard in the TIC and h is the peak height detected in the sample for that specific mass to charge ratio that corresponds to the chemical in question.

**Table 2:** Corresponding mass to charge ratio, LOD and detected level for each constituent found using LC-MS

Constituent	m/z	LOD (μg/ml)	Detected level in OSTE+ extracts (μg/ml)	Detected level in OSTE+H_2_O extracts (μg/ml)
DBN	125	2.0	37.0	31.6
D.E.N 431	163, 325–327, 488-491	n,d.^1^	n,d.	n,d.
Diallyl	215	2.0	2.4	b,d,l,^2^
TPO-L	121, 147, 317	1.2	2.4	1.4
Triallyl	250	1.7	2.4	b,d,l.
Trithiol	524	0.7	b,d,l.	b,d,l.

^1^n,d. not determined

^2^b,d,l. below detection limit

**Fig. 2:**
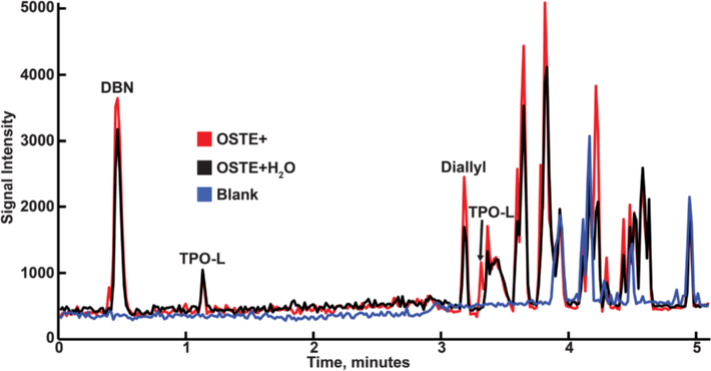
Mean TIC chromatograms from LC-MS: red is OSTE+, black is OSTE+H_2_O and the blue is the blank sample, *n* = 2. Analysis shows that one peak mainly contains DBN, two peaks that contain TPO-L and one peak contains some diallyl

TIC chromatograms from the LC-MS analysis of OSTE+ extracts, OSTE+H_2_O extracts and blank samples are shown in Fig. 2. Traces of all chemicals except trithiol were found in the OSTE+ extracts, while in the OSTE+H_2_O extracts only DBN and TPO-L were detected. It should be noted that each peak shown in Fig. 2 contains several different chemicals that contribute to the total height of the peak; hence the peak labeled “Diallyl” contains several different chemicals and diallyl is only a contributor in OSTE+, and is not found in OSTE+H_2_O. Unidentified contributors to the peak heights imply that there were impurities in the chemicals or that they are derived from the chromatographic set up. When comparing the peak height of all the different chemicals in the OSTE+ and OSTE+H_2_O extractions, the detected levels of each chemical are either lower or below LOD for OSTE+H_2_O. Acetone and D.E.N 431 were not analyzed since they are not water-soluble and standard curves could not be made.

### Chemical analysis using GC-MS

Standards of all constituents, extracts of OSTE+, extracts of OSTE+H_2_O and blank samples were analyzed using GC-MS. The standards were analyzed using the GC-MS in scan mode. Peaks and different mass to charge ratios for all chemicals were obtained from this analysis. In order to increase the sensitivity of the GC-MS, all mass to charge ratios found when running the GC-MS in scan mode were used to run the GC-MS in SIM mode. Analysis of data from running the GC-MS in SIM mode allowed for the calculation of the lower limit of detection of all chemicals (see Table 3) in the same way as described for the LC-MS.

**Table 3:** LOD of GC-MS (SIM mode)

Constituent	LOD of GC-MS
DBN	1.6 μg/ml
D.E.N 431	2.8 μg/ml
Diallyl	4.1 μg/ml
TPO-L	2.8 μg/ml
Triallyl	2.4 μg/ml
Trithiol	28.8 μg/ml

All extracts of OSTE+, OSTE+H_2_O, OSTE+/HCl, OSTE+/NaOH, OSTE+H_2_O/HCl, OSTE+H_2_O/NaOH and all blank samples were run through the GC-MS both in SCAN mode and in SIM mode. No detectable traces of the constituents were found in any of the extracts.

#### SEM analysis

**Fig. 3:**
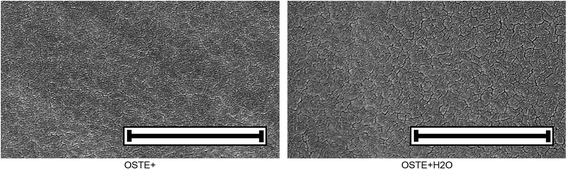
Scanning electron microscopy images of OSTE+ and OSTE+H_2_O. Scale bar = 1 μm

SEM images of OSTE+ and OSTE+H_2_O samples are shown in Fig. 3. Visible inspection revealed that the nano-sized cracks are slightly bigger in the OSTE+H_2_O than in the OSTE+ sample.

### Cytotoxicity

**Fig. 4:**
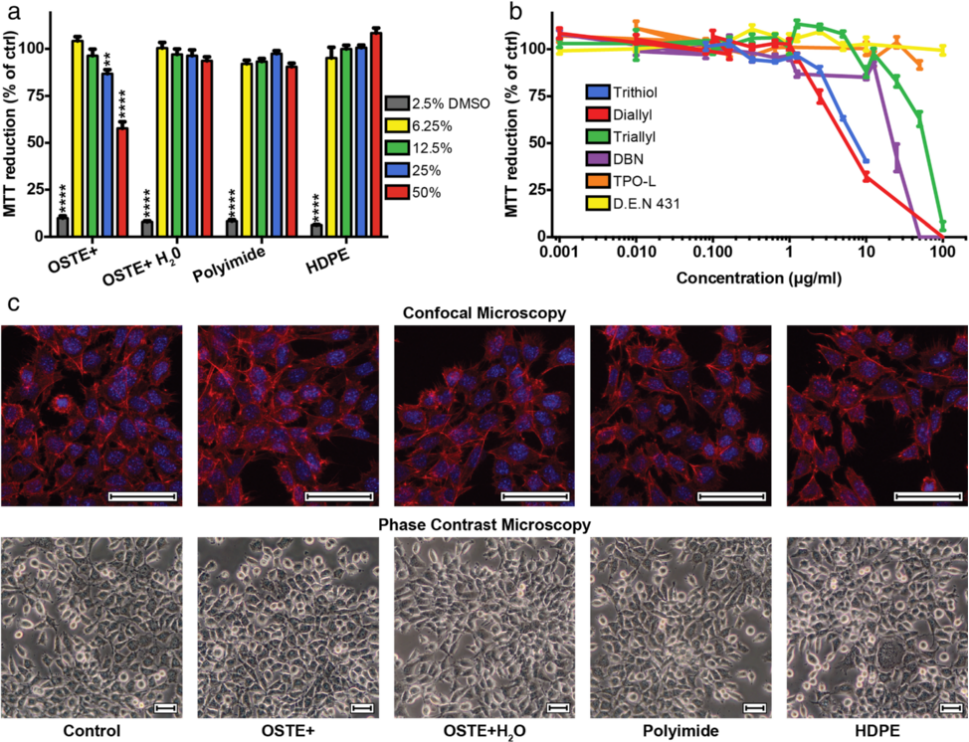
**a** Results from MTT assay tests using the L929 cell line. The L929 cells were seeded and allowed to attach in the wells of 96-well plates for 24 h. The cells were then subjected to extraction solutions of OSTE+, OSTE+H_2_O, polyimide and HDPE for 72 h at different concentrations (6 %, 12.5 %, 25 % and 50 %). DMSO at 2.5 % was used as a positive control. The results are presented as mean values (*n* = 18–36) and the error bars represent ± SEM. The data was analyzed using a 2-way ANOVA, comparing the data for each set *vs.* control where ** represents a *P*-value ≤ 0.01 and **** represents a *P*-value ≤ 0.0001. **b** The L929 cells were seeded and after 24 h treated for 72 h with the chemicals that were used in the production of OSTE+. Cell viability was assessed using the MTT assay. The results are presented as mean values (*n* = 6–18) and the bars represent ± SEM. All chemicals, used as single agents, are non-toxic at concentrations below 1 μg/ml. **c** Phase contrast (top row) and confocal microscopy (bottom row) images of mouse L929 cells exposed to extracts of OSTE+, OSTE+H_2_O, polyimide and HDPE at a 50 % concentration for 72 h. The phase contrast images show viable and non-stained cells whereas the cells visualized with confocal microscopy were fixed and stained to show the actin cytoskeleton (red) and the nuclei (blue). The scale bars are equal to 50 μm. Images shown were taken in areas with similar cell densities

To evaluate the cytotoxicity of the OSTE+ and OSTE+H_2_O extracts *in vitro*, the mouse fibroblast cell line L929 was used in MTT assays. Extracts from HDPE and polyimide were used a negative controls and references samples, respectively, and 2.5 % DMSO was used as a positive control (Fig. 4a). The L929 cells were treated with concentrations ranging from 6.25–50 %, of the different polymer extracts for a total time of 72 h. The data was analyzed using a 2-way ANOVA, comparing the data for each set *vs.* control and the results show a statistically significant and concentration-dependent decrease in MTT reduction in cells treated with OSTE+ extracts, compared to control cells (Fig. 4a). When diluted to 50 %, the OSTE+ extract lowered the MTT reduction about 40 % compared to control implying that there were 40 % fewer cells compared to control since MTT reduction is assumed to reflect the number of viable cells. At a 25 % dilution, the MTT reduction was lowered about 15 % compared to control. The same indications of toxicity seen after treatment with OSTE+ extracts were not found after treatment with OSTE+H_2_O, polyimide or HDPE extracts (Fig. 4a). Additionally, to investigate effects on cell morphology, cells treated with the different polymer extracts (50 %) were studied using phase contrast and confocal microscopy. The cells photographed in the phase contrast microscope were non-stained and non-fixed (*i.e.* viable) whereas the cells photographed in the confocal microscope were fixed and stained with phalloidin and bisbenzimide to visualize the cytoskeleton (actin filaments) and the nuclei, respectively (Fig. 4c). No changes in morphology could be seen in cells treated with the polymer extracts for 72 h, compared to control. Thus, the OSTE+ toxicity found in the MTT assay did not result in a morphological change.

To further study the toxicity of OSTE+ and to evaluate the difference in toxicity between OSTE+ and OSTE+H_2_O, the individual chemicals used to produce OSTE+ were tested in MTT assays. As seen in Fig. 4b, DBN, diallyl, trithiol and triallyl had effects on cell viability at concentrations above 1 μg/ml. IC_50_ values for all constituents were obtained using linear regression in the area around the 50 % MTT reduction (EXCEL, Table 4) and compared to the levels found when using LC-MS modified to represent extracts diluted to 50 %, *i.e.* the reported levels from Table 2 were divided by 2. DBN was the only chemical were the detected level was close to the IC_50_ level.

**Table 4:** IC_50_ values obtained from the dose response curves shown in Fig. 4b and levels detected in the LC-MS analysis modified to represent extracts diluted to 50 %

Constituent	IC_50_ (μg/ml)	50 % OSTE+ extracts (μg/ml)	50 % OSTE+H_2_O extracts (μg/ml)
DBN	23,1	18.5	15.8
D.E.N 431	>100	n,d.^1^	n,d.
Diallyl	6.8	1.2	b,d,l.^2^
TPO-L	>100	1.2	0.7
Triallyl	61.3	1.2	b,d,l.
Trithiol	7.9	b,d,l.	b,d,l.

^1^n,d. not determined

^2^b,d,l. below detection limit

## Discussion

OSTE+ is a new polymer that is more flexible then other polymers used for neural probes. This should allow probes constructed in OSTE+ to follow the micro motions of the brain, hence increasing the biocompatibility of the neural probe [16]. Our group has previously shown that it is possible to make implantable neural probes in OSTE+ [18]. However there is one important question: is OSTE+ biocompatible? In our study we decided to give an answer to this question by using standardized biocompatibility toxicity tests used by the industry. Cytotoxicity was investigated using MTT assays with L929 cells in compliance with ISO standard 10993–5. The MTT assay was used to investigate the cytotoxicity of extracts of four different materials (OSTE+, OSTE+H_2_O, polyimide and HDPE) and of the individual chemicals that were used in the OSTE+ fabrication process. The morphology of cells treated with extracts was investigated using phase contrast and confocal microscopy. GC-MC and LC-MS were used to analyze chemicals found in extracts of OSTE+ and extracts of OSTE+H_2_O. SEM was used to investigate the surface structure of OSTE+ and OSTE+H_2_O.

The results of the cytotoxicity test (Fig. 4a) imply that OSTE+ used directly after production may be toxic if not washed properly before implantation. The reduction of MTT is assumed to be proportional to cell number and thus at the highest concentration (extract diluted 50 %), the OSTE+ extract significantly reduced the cell number thus implying an effect on cell proliferation. Using confocal microscopy and phase contrast microscopy we could not find any morphological changes imposed by any of the treatments (Fig. 4c). We cannot, however, exclude the possibility that higher magnification microscopy or the use of other cellular staining probes than phalloidin staining of actin may indicate morphological changes. To be able to draw conclusions about the exact mechanism of toxicity, more elaborate investigations have to be made. On the other hand, that may not be warranted since the toxicity disappeared when the OSTE+ samples were pre-incubated in water as an extra washing step.

The chemical analysis (LC-MS) showed that the water-soluble chemicals DBN, diallyl, triallyl and TPO-L were found in the extracts made from OSTE+. But only DBN and TPO-L were detected in the OSTE+H_2_O extracts, which is expected since they are only used as initiators in the process and are not immobilized in the final polymer network. Because the detected levels of most constituents were close to the respective LOD, some may still have leached out into the OSTE+H_2_O extracts. The IC_50_ value found for DBN (Table 4) is close to the detected levels of DBN in extracts of OSTE+ and in extracts of OSTE+H_2_O, and as seen in Fig. 4b the steep curve for DBN could explain the difference in toxicity between the extracts OSTE+ and the extracts of OSTE+H_2_O. Comparing the IC_50_ values for diallyl and triallyl in the OSTE+ extracts with the detected levels (Table 4) the IC_50_ values are six times greater for diallyl and 60 times greater for triallyl. Hence, our conclusion is that neither diallyl nor triallyl are responsible for the toxicity found in the OSTE+ extracts. The lack of results from the extracts analyzed using the GC-MS is probably because most of the constituents are water soluble and thus were not extracted into the toluene phase from the water phase. Since acetone was used in the fabrication process, it is a possible candidate for toxicity. However, acetone was used as a 3 % concentration in the fabrication mixture which was then degassed, exposed to UV-light and baked at 65 °C over night. Since acetone is a highly volatile substance, the concentration was likely lowered substantially. Remaining acetone may of course have leaked into the extract but a hypothetical calculation of concentration in the extract with no evaporation during the production process would give at hand a maximum concentration in the medium of 0.2 % which is below a concentration that has shown toxicity in cells [30, 31]. Thus, we do not think acetone contributed to toxicity of OSTE+. The unidentified peaks in the LC-MS spectra (Fig. 2) support the existence of impurities, but since we were unable to identify the peaks their role in the toxicity cannot be determined. Neither have we tested possible synergistic effects of the low doses of the leaked compounds. Since we did not autoclave the samples used in the chemical testing, there could be a small variation between the extracts used in the MTT assays and in the chemical testing. However, the variation should be insignificant since the state of the material does not change when increasing the temperature above the temperature used in the hard-baking step.

Since oxygen will inhibit the UV-initiated polymerization step and PDMS has a high oxygen permeability [32, 33], the use of a spin-casting technique instead of PDMS molds should allow for a better polymerization of OSTE+ and decrease the amount of unreacted constituents in the final polymer. The surface analysis, using SEM (Fig. 3) shows that there was a small difference between OSTE+ and OSTE+H_2_O samples. The nano-sized cracks were somewhat wider in the OSTE+H_2_O sample. In our previous work we did not use acetone in the mixture and we did not see any cracks when using that formula [18]. We therefore hypothesize that these cracks appeared during a fabrication step where acetone evaporated. Since acetone was added in order to make the mixture less viscous and easier to use in a mold, the use of spin casting should allow for a fabrication protocol without the use of acetone, which hopefully will prevent the formation of nano-sized cracks.

## Conclusion

OSTE+ is a very promising material to use for neural probes because of its superior flexibility compared to other polymers used in neural probes today. The toxicity of OSTE+ extracts was presumably derived from DBN, unknown impurities in the constituents or from low dose synergetic effects of the constituents. However, by pre-incubating the final polymer in water for 7 days, OSTE+ was shown to be nontoxic to cells. The use of high-purity chemicals in the fabrication process, removal of acetone from the fabrication, the addition of a cleaning step in water during fabrication and the use of a silicon wafer as the substrate instead of PDMS should minimize the toxicity rendering implants made from OSTE+ nontoxic. In order to ensure the biocompatibility of OSTE+ for neural implants both short and long term *in vivo* tests have to be conducted.
